# Protein kinase C signaling “in” and “to” the nucleus: Master kinases in transcriptional regulation

**DOI:** 10.1016/j.jbc.2024.105692

**Published:** 2024-01-30

**Authors:** Marcelo G. Kazanietz, Mariana Cooke

**Affiliations:** Department of Systems Pharmacology and Translational Therapeutics, Perelman School of Medicine, University of Pennsylvania, Philadelphia, Pennsylvania, USA

**Keywords:** protein kinase C, signal transduction, nucleus, gene expression, transcription factor, diacylglycerol

## Abstract

PKC is a multifunctional family of Ser-Thr kinases widely implicated in the regulation of fundamental cellular functions, including proliferation, polarity, motility, and differentiation. Notwithstanding their primary cytoplasmic localization and stringent activation by cell surface receptors, PKC isozymes impel prominent nuclear signaling ultimately impacting gene expression. While transcriptional regulation may be wielded by nuclear PKCs, it most often relies on cytoplasmic phosphorylation events that result in nuclear shuttling of PKC downstream effectors, including transcription factors. As expected from the unique coupling of PKC isozymes to signaling effector pathways, glaring disparities in gene activation/repression are observed upon targeting individual PKC family members. Notably, specific PKCs control the expression and activation of transcription factors implicated in cell cycle/mitogenesis, epithelial-to-mesenchymal transition and immune function. Additionally, PKCs isozymes tightly regulate transcription factors involved in stepwise differentiation of pluripotent stem cells toward specific epithelial, mesenchymal, and hematopoietic cell lineages. Aberrant PKC expression and/or activation in pathological conditions, such as in cancer, leads to profound alterations in gene expression, leading to an extensive rewiring of transcriptional networks associated with mitogenesis, invasiveness, stemness, and tumor microenvironment dysregulation. In this review, we outline the current understanding of PKC signaling “in” and “to” the nucleus, with significant focus on established paradigms of PKC-mediated transcriptional control. Dissecting these complexities would allow the identification of relevant molecular targets implicated in a wide spectrum of diseases.

## PKC isozymes: key regulators of signal transduction

The transmission of extracellular signals leading to cellular responses is largely initiated by cell surface receptors, protein sensors for chemical or physical inputs that elicit intracellular biochemical events such as protein phosphorylation, to spark the modulation of signal transduction cascades. In multicellular organisms, coordinated signaling steps involving kinases and phosphatases are central to the control of basic cellular functions, including cell division, metabolism, and motility, as well as contribute to the regulation of cell morphology and fate. These complex mechanisms rely on events occurring with fast kinetics, namely posttranslational modifications and relocalization of proteins and others happening at slower rates, such as those involving the transcription of genes that undeniably require signal transmission to the nucleus.

Among the multiple receptor-dependent signaling players, PKC embodies one of the utmost studied enzyme families responsible for the control of vital cellular processes *via* phosphorylation. PKC epitomizes the archetypal lipid–regulated kinase. Members of the PKC family are recognized as the main intracellular receptors for the lipid second messenger 1,2-diacylglycerol (DAG), which together with inositol-1,4,5-triphosphate comprise the products of phosphatidylinositol-4,5-biphosphate breakdown by phospholipase C (PLC). PLCs become primarily activated in response to G protein–coupled receptor or receptor tyrosine kinase stimulation, which results in the rapid and transient generation of DAG together with inositol-1,4,5-triphosphate–mediated rise in intracellular calcium. The PKC family comprises three classes of Ser/Thr kinases classified according to their distinct biochemical regulation: the DAG/calcium sensitive “classical/conventional” PKCs (cPKCs α, βΙ, βΙΙ, and γ), the DAG-sensitive, calcium-insensitive “novel” PKCs (nPKCs δ, ε, η, and θ) and the DAG/calcium-insensitive “atypical” PKCs (aPKCs ζ and ι). The latter family lacks essential structural elements for binding DAG and calcium, and they are uniquely regulated *via* phosphorylation and protein–protein interactions (1, 2, 3, 4).

**Figure 1:**
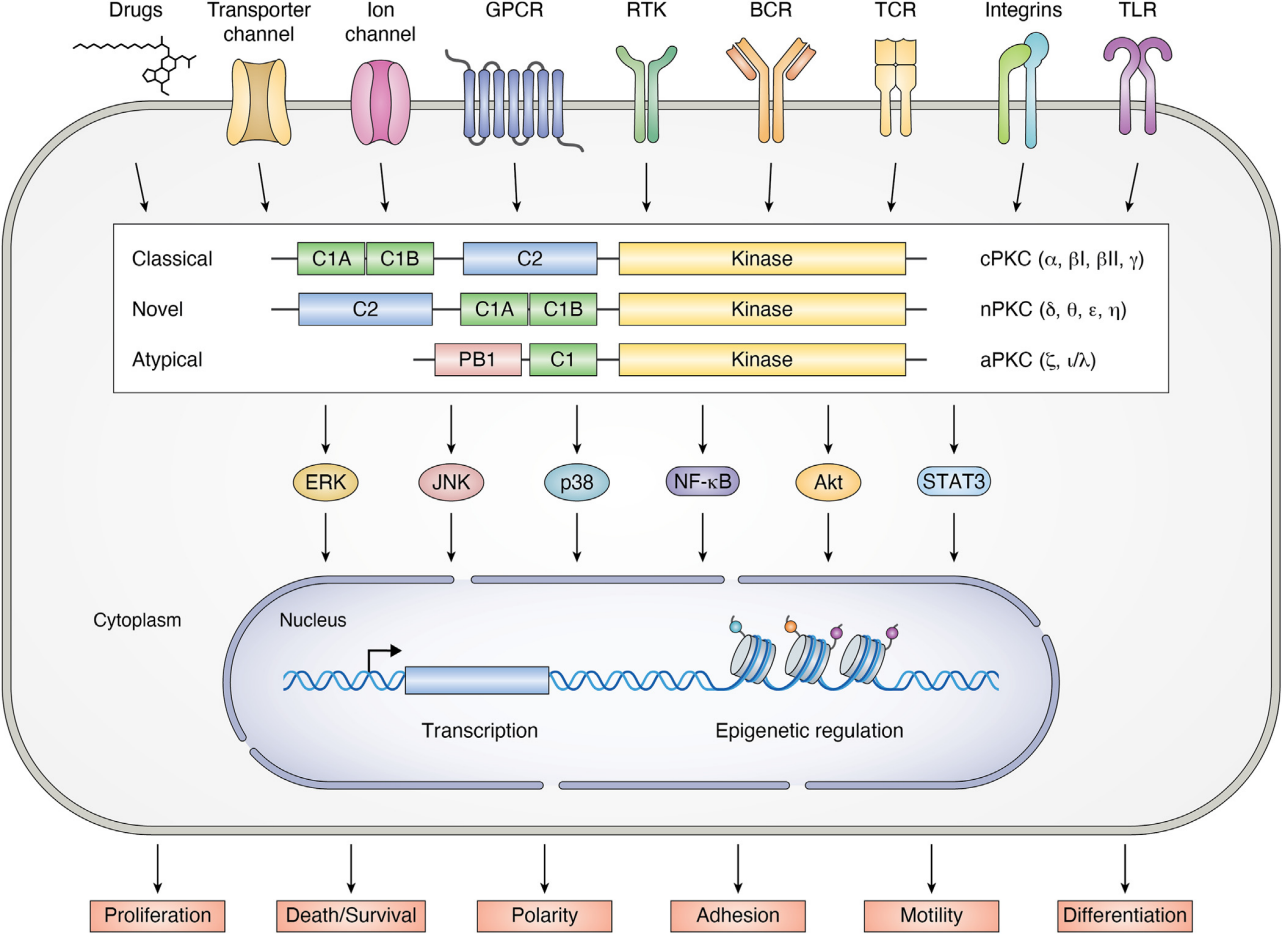
**PKC isozymes and signal transduction.** PKCs are classified into classical/conventional, novel, and atypical based on their distinctive biochemical and structural properties. Cell surface receptors or drugs can trigger the activation of discrete PKCs, which phosphorylate many cellular effectors, including signal transduction proteins that impact nuclear function. BCR, B cell receptor; GPCR, G protein–coupled receptor; RTK, receptor tyrosine kinase; TCR, T cell receptor; TLR, toll-like receptor.

From a structural standpoint, PKC isoforms hold well-defined N-terminal regulatory and C-terminal catalytic regions linked by a hinge region or V3 domain (Fig. 1). Within the regulatory region, both cPKCs and nPKCs have two 50 amino acid long C1 domains (C1A and C1B) with DAG binding capabilities and a C2 domain involved in phospholipid binding. The C2 domain also accounts for calcium binding in cPKCs, but it lacks calcium binding competence in nPKCs. The C-terminal catalytic region contains the ATP binding site in the C3 domain and the C4 domain responsible for phosphotransferase activity (1, 2, 3, 4, 5). The newly synthesized PKC undergoes a series of phosphorylation events and adopts an autoinhibited conformation through intramolecular interactions, such as the interaction of the pseudosubstrate region in the N terminus and the active site in the C-terminal kinase domain. Further intramolecular interactions between the C1b domain and an “NFD” motif in the catalytic region contribute to the autoinhibited conformation, with amino acids upstream this motif filling the DAG binding site of the C1 domain. cPKCs and nPKCs share a common allosteric activation mechanism by DAG, as extensively demonstrated using biochemical and structural approaches. Full enzyme activation by DAG or DAG-mimicking agents, such as the phorbol esters, requires the destabilization of both autoinhibitory interfaces. The accepted model for cPKC activation suggests that the C2 domain engages with the membrane after calcium binding, followed by C1A domain binding to DAG for stabilization of membrane association and C1B membrane binding once unclamped from the NFD domain (4, 5, 6, 7, 8, 9, 10).

A large body of literature shows that individual members of the PKC family associate with unique subsets of cellular responses; nonetheless, overlapping PKC isoform functions have been also broadly described. PKC isozyme functional selectivity (or lack thereof) is likely multifactorial and includes the distinctive (or concurring) modes of regulation by activators and cofactors, as well as unique (or similar) substrate specificity. However, a main driver for selectivity is their competence to localize to specific intracellular compartments upon activation, for example, the plasma membrane (where DAG is largely generated in response to stimulation of PLC-coupled surface receptors), nuclear membrane, endoplasmic reticulum, Golgi, and mitochondria. Notably, DAG-mimicking agents can induce the translocation of cPKC and nPKC isozymes to different intracellular compartments. Precise subcellular localization is presumably dictated by specific associations with protein and lipids, ultimately leading to preferential phosphorylation of effectors and functional specialization (4, 11, 12, 13, 14, 15, 16). It would be fair to state that, at the present time, the aforementioned model for PKC activation does not fully contemplate protein–protein interactions at confined cellular regions or additional regulatory mechanisms such as posttranslational modifications, for example, Tyr phosphorylation (17, 18, 19, 20). Another level of complexity given their unique biochemical regulation and localization is the diversity of downstream signaling controlled by each member of the PKC family. PKC isozymes are considered major nodes for a vast array of signaling pathways, including MEK/ERK, JNK, p38, and NF-κB among others (21, 22, 23) (Fig. 1). Secondary waves of phosphorylation events driven by PKC effector kinases ultimately result in large phosphoproteome signatures that comprise both direct and indirect PKC phospho-substrates.

A remarkable trait of PKCs is their ability to carry out nuclear signaling and impact the transcriptional regulation of genes. Consequently, PKC activation has a profound influence over the control of gene expression and transcriptional networks. This feature becomes evident in scenarios of aberrant PKC expression and/or PKC activation, as observed in cancer, which results in deregulated transcriptional activation/repression of oncogenic networks associated with the transformed phenotype.

This review primarily focuses on the mechanisms by which PKC isozymes control signal transduction pathways responsible for nuclear function. Emphasis will be placed on well-established paradigms of PKC-mediated transcriptional control that involve either PKC nuclear localization or PKC effector pathways that ultimately effect nuclear events.

## PKC and transcriptional regulation: a bit of history

The earliest link between PKC and nuclear function was established in the 1980s, at a time when the PKC genes and their corresponding isozyme products had not yet been identified. Seminal work by Michael Karin (24) found several genes that could be transcriptionally induced by phorbol esters, leading to the identification of the “12-tetradecanoyl phorbol 13-acetate (TPA)-responsive element” (TRE). The TRE, originally characterized by footprint analysis in the promoter of the human collagenase gene, represents the minimal element sufficient for induction by TPA, also known as phorbol 12-myristate 13-acetate or PMA). These studies, which paralleled the identification of cyclic AMP response element binding protein as the PKA-responsive element (25), established TRE as the binding site for the nuclear factor activator protein-1 (AP-1), later characterized as a noncovalent complex between proto-oncogenes c-fos and c-jun. The increased binding of AP-1 to the palindromic TRE sequence TGA(C/G)TCA in TPA/PMA-treated cells is caused by *c-fos* and *c-jun* induction, as well as posttranslational modifications of these oncoproteins (24, 26, 27, 28, 29). Subsequent studies in *Ha-Ras* transformed cells mapped phosphorylation on Ser63 and Ser73 in c-Jun as a requisite for the activity of this transcription factor (30). The identification of AP-1 as a “final” downstream target of the PKC signal transduction pathway constituted the first functional link between PKC activation and transcriptional activation of genes.

A second breakthrough linking PKC and gene transcription was the discovery of NF-κB, initially identified as a nuclear protein within the lymphoid lineage that interacts with the κ immunoglobulin light chain enhancer (31). David Baltimore’s lab demonstrated that PMA causes a striking induction of NF-κB, an effect that depends on the posttranslational modification of a preexisting protein (32). His laboratory also reported the dissociation of the cytoplasmic NF-κB/inhibitor of κB (IκB) complex in response to phorbol ester treatment. Phosphorylation of IκB turned out to be the critical event for the release of active NF-κB from this complex, resulting in NF-κB relocalization into the nucleus where it activates specific target gene enhancers (33, 34).

After the cloning of PKC genes and the initial delineation of the roles of individual PKCs as promoters or suppressors of malignant transformation (see below), it became evident that PKC isozyme-specific regulation of transcription takes place. Initial studies carried out by Ohno and co-workers found dissimilar effects for each the three major DAG-responsive PKC isoforms expressed in fibroblasts, PKCα, PKCδ, and PKCε, on the activation of a reporter plasmid containing binding sites for the transcription factor E2F, which are commonly found in promoter regions of G1/S cell cycle genes (35). This finding was in consonance with the reported bimodal regulation of E2F and DNA synthesis by phorbol esters, as well as with the distinctive roles of individual PKCs in different phases of the cell cycle (18, 36, 37, 38, 39, 40, 41). Despite the shortcomings of overexpressing approaches used in this early study, these results provided proof-of-principle for the unique functional involvement, including opposite roles, of PKC isozymes in transcriptional control. Subsequent studies by the Weinstein group using dominant-negative and constitutively active PKC mutants recognized a differential involvement of individual PKCs in activation of the *c-fos* serum response element (42). Promoter reporter assays for discrete genes, including *cyclin A*, *cyclin D1*, and *p21*^*Cip1*^, became instrumental for underscoring eminent PKC isozyme-specific links with cell cycle progression (39, 40, 43, 44, 45).

Our laboratory carried out the first unbiased genome-wide study that led to the identification of PKC isozyme-specific transcriptional signatures in cellular models (46). A longitudinal analysis of PMA-regulated genes in LNCaP prostate cancer cells revealed major changes in gene expression at early time points, followed by secondary waves of transcriptional changes likely driven by immediate-early transcription factors induced by PKC activation. Major differences in the expression of early genes, both gene induction and repression, were found among cells subjected to RNAi for PKCα, PKCδ, or PKCε, with minimum overlapping in genes regulated by all three PKCs. In this cellular model, PKCδ turned out to be the most prominent isozyme controlling gene expression, particularly for the regulation of apoptotic-related genes. Opposite roles were observed for PKCδ and PKCε, a finding consistent with the contrasting roles of these nPKCs in apoptosis/survival and mitogenesis/antimitogenesis (see below). Gene enrichment analysis identified over-representation of the responsive element *c-Rel*, a member of the NF-κB family (47), in the promoters of PKCδ-regulated genes. Moreover, significant interconnections were established between PKCδ-regulated genes *via c-Rel* and specific functions, including angiogenesis, inflammation, and cell motility. Interestingly, silencing *c-Rel* sensitizes prostate cancer cells to PMA-induced apoptosis, corroborating the involvement of *c-Rel* as a driver of prosurvival transcriptional programs in prostate cancer cells (48).

A second unbiased genome-wide analysis turned out to be instrumental for identifying PKC isozyme-specific gene expression signatures in lung cancer cells (49). Major enrichment in NF-κB binding sites among the regulated genes as well as associations with NF-κB–related functions, namely cytokine production, could be established. A PKCα-specific program for metalloprotease gene expression was particularly evident, which provided a functional link with extracellular matrix degradation. The glaring disparities in transcriptional networks controlled by individual PKC isozymes in distinct cellular models are a strong indicator of exclusive regulation depending on the context.

## PKC signaling: “in” or “to” the nucleus?

**Figure 2:**
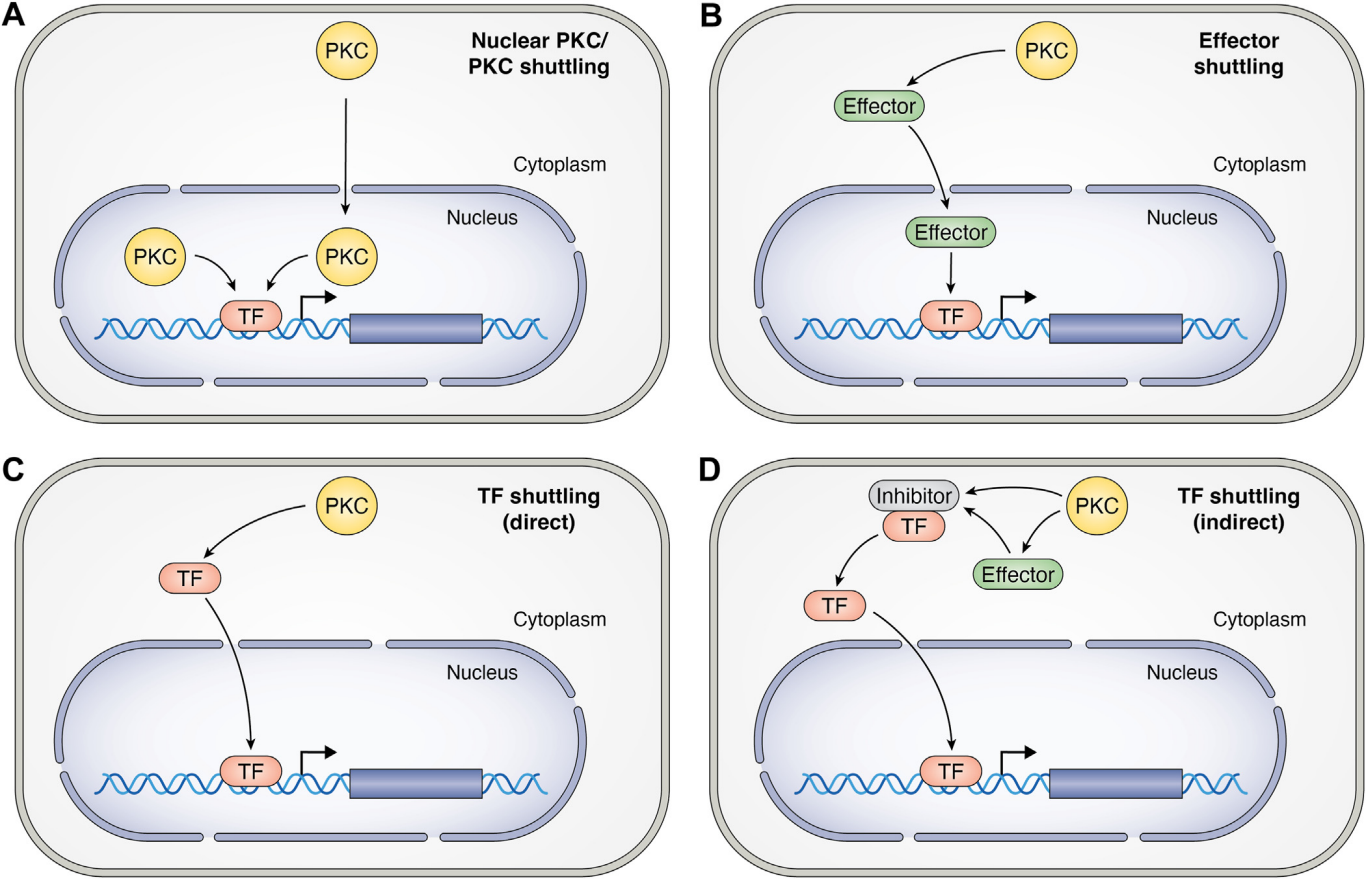
**Hypothetical models for PKC signaling driving nuclear function.** The *cartoo*n depicts different models for transcriptional control by PKC. *A*, PKC isozymes can be either located in the nucleus or translocated to the nucleus, where they phosphorylate components of the transcriptional complexes, including transcription factors (TFs), and turn on or off the transcriptional activation of selected genes. *B*, PKCs can regulate transcription *via* their downstream effectors, which shuttle to the nucleus upon phosphorylation by PKC or PKC effector kinases. *C*, PKCs localize in the cytoplasmic compartment and upon activation phosphorylate TFs, which in turn shuttle to the nucleus. *D*, PKCs (or PKC effector kinases) can phosphorylate proteins that bind (and inhibit) transcription factors. Phosphorylation of these inhibitory proteins in the complex leads to the dissociation of the transcription factor and its translocation to the nucleus.

PKC isozymes largely localize in the cytoplasm, either in the soluble cytosolic compartment or associated to internal membranes (4, 11, 12, 13, 14, 15, 16). A potential model for PKC-mediated regulation of nuclear functions entails the reliance on PKC pools located in the nuclear compartment. Alternatively, it could involve PKC isozyme cytoplasmatic-nuclear shuttling or the phosphorylation of cytoplasmatic substrates capable of modulating nuclear function (Fig. 2). Notwithstanding, the broad literature on PKC nuclear compartmentalization is quite controversial. While early studies have reported PKC localization in the nucleus or PKC association with nuclear/perinuclear membranes (50, 51, 52, 53, 54), the inherent limitations of subcellular fractionation assays and immunofluorescent approaches could lead to artifactual results, thus conclusions should be interpreted with great caution.

For the most part, PKC nuclear localization studies lack reliable evidence for correlative functional associations. A notable exception has been reported for the proapoptotic PKCδ, studies that were pioneered by the Reyland laboratory. It has been thoroughly described that caspase-mediated proteolytic PKCδ cleavage, occurring at the V3 hinge region, takes place upon subjecting cells to ionizing radiation or by treatment with apoptosis inducers such as DNA-damaging agents. The apoptotic agents trigger the sequential phosphorylation of PKCδ by soluble tyrosine kinases, namely c-Abl and Src, leading to a conformational change that exposes a C-terminal nuclear localization signal likely occluded in the autoinhibited state, followed by binding to importin and nuclear transport. Heat shock protein 90 binds to PKCδ with similar kinetics as importin, possibly facilitating importin binding to the PKCδ C-terminal domain. Once in the nucleus, PKCδ is cleaved by caspase-3, leading to the generation of a constitutively active C-terminal PKCδ catalytic (kinase) fragment. Notably, a nuclear localization signal PKCδ mutant is unable to shuttle to the nucleus and fails to trigger an apoptotic response (18, 55, 56, 57, 58, 59, 60). Nuclear PKCδ likely phosphorylates cell death–related substrates and activates apoptotic transcriptional programs. A recognized target for PKCδ in response to DNA-damaging agents is STAT1, which becomes phosphorylated on Ser727, suggesting that PKCδ-mediated regulation of apoptotic responses involves the activation of STAT1 target genes (61). PKCδ also associates with and phosphorylates nuclear proteins involved in DNA processes, such as topoisomerase IIβ and Rad9 (62, 63), and transcription factors such as RelA/p65 NF-κB (64, 65). Very recently, PKCδ was found to exert prominent effects on nonhomologous end joining and homologous recombination–mediated DNA double-strand break repair, chromatin remodeling, and histone methylation (66). Additional examples of functionally relevant relocalization of DAG-responsive PKCs to the nucleus/nuclear envelope include the PKCβII-mediated phosphorylation of mitotic lamin B (67, 68), PKCα-mediated lamin B disassembly (69), and PKCθ association with chromatin (70, 71, 72). Still, whether allosteric activation of PKCs occurs in the nuclear compartment remains to be determined. It is important to emphasize that specific members of the PLC family responsible for DAG generation and DAG kinases responsible for DAG phosphorylation to phosphatidic acid have been found in the nucleus and in specific cases functionally associated with responses such as cell cycle control, proliferation, and differentiation (73, 74, 75, 76, 77, 78, 79, 80). Most recently, a study in lower eukaryotes reported that DAG levels at the inner nuclear membrane are dynamically regulated during mitosis, arguing that DAG effectors such as PKC may sustain nuclear signal transduction (81). The presence of nuclear import and export signals in PKCι suggests DAG/calcium-independent nuclear PKC signaling (82).

Nonetheless, the consensus is that most nuclear functions controlled by PKCs depend on extranuclear phosphorylation events rather than intranuclear activation of PKC, involving phosphorylation by PKC itself or by a PKC effector kinase. In a few instances, it entails PKC-induced dissociation of extranuclear complexes between transcription factors and their inhibitory proteins, such as the NF-κB/IκB complex, ultimately leading to the nuclear shuttling of the transcription factor and transcriptional activation of target genes (33, 34). It is worth mentioning that consistent with the challenge of identifying direct PKC phosphorylation substrates (discussed in (11)); there is scant information on direct PKC phosphorylation sites on transcription factors. Additionally, as also mentioned above, the induction of specific transcription factors, such as c-fos, c-jun, could take place by activated PKC signaling (24, 26, 27, 28, 29), thus underscoring a complex array of mechanistic routes ensuing a fine-tuned control of gene expression by PKC isozymes.

The next sections will focus on representative examples of PKC-driven signal transduction mechanisms impinging on nuclear responses, with significant emphasis on gene expression programs associated with specific cellular functions.

## PKC signaling and the control of mitogenic transcriptional programs

**Figure 3:**
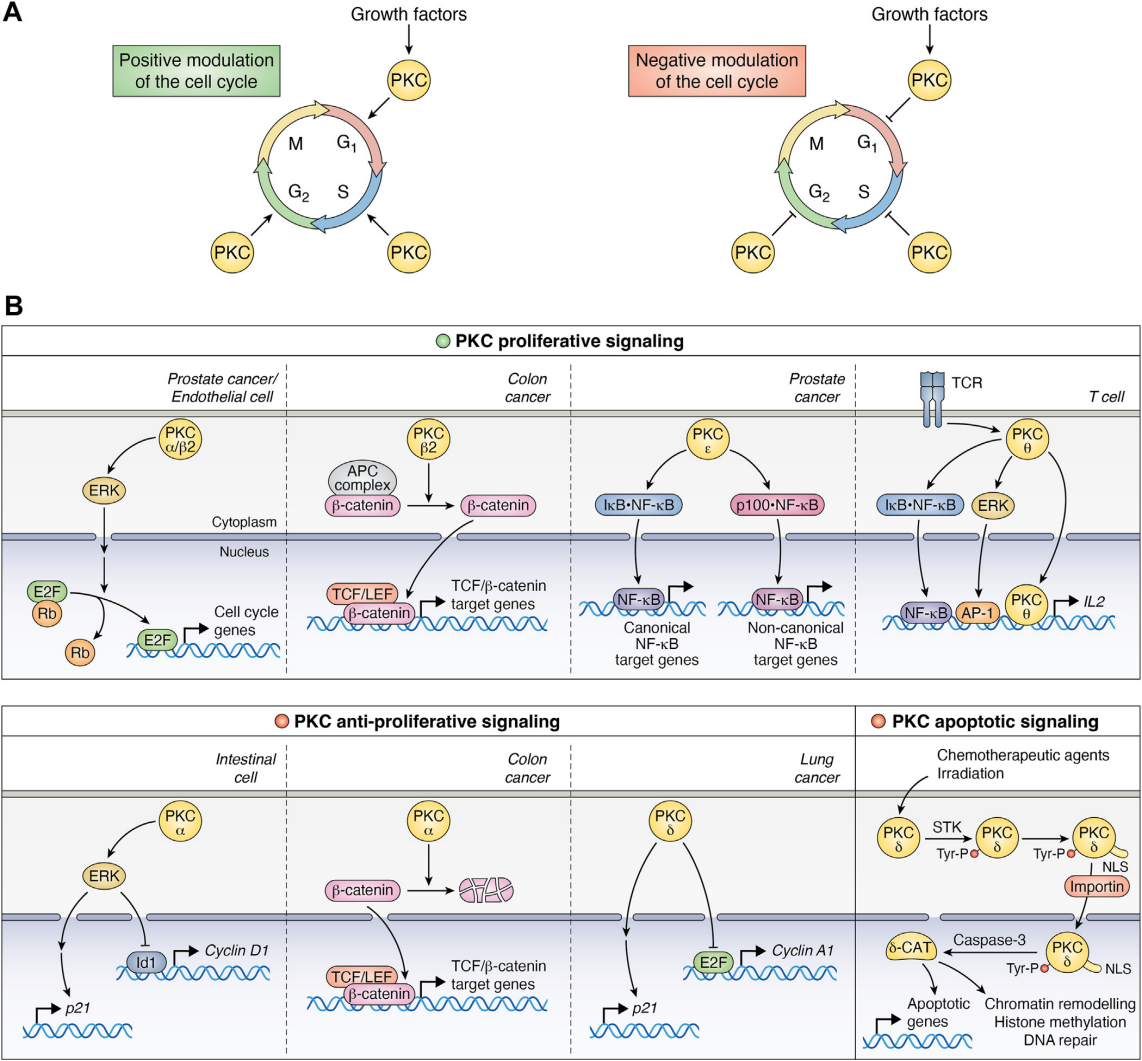
**PKC regulation of nuclear function in the control of proliferation and apoptosis.** PKC isozymes exert proliferative/prosurvival and antiproliferative/apoptotic roles in distinct cell contexts. *A*, the dual roles of PKC isozymes in proliferation are indicative of either positive (*upper panel*) or negative (*lower panel*) control of the cell cycle, acting in different phases with characteristic isozyme and cell type specificity. *B*, representative examples for positive (*upper panels*) and negative (*lower panels*) controls of nuclear events by PKC isozymes. The *lower panel* also includes an established paradigm for PKCδ in apoptotic signaling in response to chemotherapeutic drugs and irradiation. These stimuli promote PKCδ phosphorylation by soluble tyrosine kinases (STKs), exposure of a cryptic NLS, binding to importin, cytoplasmatic-nuclear shuttling, and nuclear cleavage by caspase-3, leading to the generation of an active PKCδ catalytic fragment (*δ-CAT*). NLS, nuclear localization signal.

The recognition of PKCs as the main cellular receptors for the phorbol ester tumor promoters followed decades of investigation on their contribution to proliferative and oncogenic signaling. Early reports using mouse models of skin carcinogenesis recognize that long-term phorbol ester topical application promotes the clonal expansion of initiated cells (*i.e.*, mutated by the action of a carcinogen) (83). While several studies showed that phorbol esters could act as mitogenic agents in selected cellular models (see for example Refs. (84, 85, 86)), others revealed that these agents restrict proliferation or promote apoptosis (4, 87, 88, 89, 90, 91, 92, 93, 94). This functional dichotomy became to be mechanistically untangled with the advent of pharmacological and molecular tools capable of dissecting PKC isozyme specificity and further corroborated by means of genetically engineered mouse models evidencing that discrete PKCs could have either pro-oncogenic or antitumorigenic activities (4). This paradigm is epitomized by the demonstrated antipromoting role of PKCδ and the oncogenic role of PKCε in the dimethylbenz[a]anthracene/TPA-induced skin tumorigenesis mouse model (4, 95, 96, 97, 98). The demonstration of antiproliferative/apoptotic and proliferative/prosurvival effects by these kinases in cellular models, respectively, further supports this duality (40, 41, 99, 100, 101, 102, 103, 104, 105). There is compelling evidence for a tight control of cell division by individual PKC isozymes, acting either as positive or negative regulators of the cell cycle, as described below. Many of the PKC-driven cell cycle–dependent regulatory steps are mediated by signaling cascades impacting on transcription factors that contribute to either activation or suppression of cell proliferation, as shown in Figure 3.

### PKC isozymes as positive regulators of proliferation and tumorigenesis

PKCs have been widely characterized as growth promoting kinases in restricted physiological and pathological settings. Studies established the involvement of the DAG-generating enzyme PLCγ1 in mitogenic responses by growth factors, including epidermal growth factor, platelet-derived growth factor, and vascular endothelial growth factor (106, 107, 108, 109). The Raf/MEK/ERK kinase cascade was identified as a primary effector of PKC mitogenic signaling. For example, the mitogenic activity of VEGF in endothelial cells is mediated by a PKCβ2–ERK axis that enables the progress from G1 to later stages of the cell cycle. This action is mediated by enhanced retinoblastoma protein phosphorylation and the subsequent dissociation of Rb–E2F complexes leading to increased E2F activation (110).

Allegedly, the mitogenic activity of PKCs is largely isozyme- and context-dependent. For example, mitogenic signaling in T lymphocytes has been widely linked to PKCθ. Upon contact with antigen-presenting cells (APCs), PKCθ in T cells translocates to the immunological synapse (IS), a site where the highly ordered signaling complexes of T cell receptor (TCR) and coreceptors localize. This effect is mediated by DAG generated by PLCγ. At the IS, PKCθ integrates signaling cascades that operate *via* the activation of transcription factors AP-1 and NF-κB to transcriptionally activate the interleukin-2 (IL2) gene and stimulate the production of this essential cytokine for T cell proliferation (111, 112, 113). PKCθ-deficient T cells have a manifest reduction in AP-1 activation in response to TCR stimulation or PMA treatment (114). The NF-κB activation induced by PKCθ in T cells is mediated by IκB kinase β (115, 116). It has been also reported that PKCθ translocates to the nucleus where it tethers to chromatin and forms a complex with RNA polymerase II, histone kinase MSK-1, and 14-3-3ζ. PKCθ redistribution to the nucleus correlates with the active transcription of genes induced upon T cell activation. Moreover, PKCθ coresides with RNA polymerase II on the promoter of inducible immune response genes to positively regulate their expression and forms chromatin anchored complexes that negatively regulate genes encoding transcriptional repressor proteins through microRNA-regulated processes (70, 72, 117). Thus, the accepted paradigm is that PKCθ fine-tunes gene expression in T cells both by initiating transcription and through the expression regulation of repressor proteins. It has been speculated that nuclear PKCθ catalytic activity helps retain RelA/p65 NF-κB in the nucleus to directly influence chromatin accessibility at transcriptional memory T cell genes (118).

Positive associations between PKCs and proliferative responses have also been extensively described in cancer. An established link between PKCβII and hyperproliferation in the colonic epithelium denotes roles for this cPKC in early steps of colon cancer, particularly due to the increased PKCβII expression observed in preneoplastic lesions and colon tumors. The proposed model is that PKCβII-mediated colon carcinogenesis involves the phosphorylation (and inactivation) of kinase glycogen synthase kinase-3β, leading to dissociation of the APC/β-catenin complex and the resultant T-cell factor (Tcf)/lymphoid enhancer factor–dependent transcription of growth promoting genes (119, 120). Another relevant example is PKCε, a pro-oncogenic nPKC that is highly expressed in solid tumors. The aberrant overexpression of PKCε has been causally associated with the growth of primary tumors as well as with cancer cell dissemination to metastatic sites (21, 23, 121, 122, 123, 124, 125, 126). Studies revealed that ectopic expression of PKCε in LNCaP prostate cancer cells stimulates proliferation, and upregulates cyclins D1, D3, and E. PKCε overexpression also causes a discernible upregulation in E2F-1 and c-myc, an oncogene with functional E2F binding sites in its promoter. High levels of phosphorylated Rb can be also observed in this setting. The accelerated transit of PKCε-overexpressing cells through the cell cycle G1 restriction point is mediated by the MEK/ERK cascade (127). Whereas PKCε tumorigenic function does not seem to rely primarily on transcriptional mechanisms in nonsmall lung adenocarcinoma (128), this kinase activates tumorigenic transcriptional programs in models of prostate cancer, particularly in cooperation with the loss of the tumor suppressor phosphatase and tensin homolog (PTEN) (a common genetic alteration in prostate cancer) (129). Global transcriptome profiling of PKCε-overexpressing/Pten deleted prostate epithelial cells revealed major changes in gene expression, particularly the activation of transcriptional programs associated with proliferation, angiogenesis, and metabolism. PKCε transcriptionally controls the expression of cytokines and eicosanoids that are prominent regulators of the tumor microenvironment mostly through the NF-κB pathway. Quite remarkably, the tumorigenic activity of prostate cancer cells driven by PKCε in conjunction with the PI3K pathway, which is activated upon PTEN loss, largely depends on both canonical and noncanonical NF-κB pathways (127, 129). The functional relevance of the PKCε–NF-κB signaling connection is also relevant in endothelial function, inflammation, diabetes, and metastasis (129, 130, 131, 132, 133, 134).

Recently, our laboratory identified PKCα as a central node for tumorigenic transcriptional networks in human prostate cancer (135). Aberrant overexpression of PKCα is a hallmark of several aggressive cancers, including prostate and triple-negative breast cancer (TNBC) (135, 136, 137, 138). Silencing PKCα expression from aggressive prostate cancer cell lines confers slower growth properties, with accumulation of cells in G0/G1, reduction in DNA synthesis, and Rb dephosphorylation. PC3 prostate cancer cell xenograft studies in nude mice revealed a major PKCα dependency for tumor growth. Notably, PKCα-depleted tumors are smaller in size and exhibit reduced phospho-ERK staining and mitotic index (*i.e.*, Ki67 staining). Characterization of the PKCα transcriptome in PC3 cells revealed a prominent enrichment in pathways associated with cell cycle progression and DNA replication, including PKCα-mediated control of mitotic cell cycle processes, chromosome segregation, and G1/S specific transcription. Most remarkably, transcription factor enrichment analysis of PKCα-regulated genes identified an E2F signature, a finding consistent with the enhanced E2F activity resulting from Rb dephosphorylation in PKCα-silenced PC3 cells (135). Altogether, these studies imply a strong association between PKCα-driven transcriptional programs and proliferative responses in aggressive prostate cancer models. Presumably, a similar scenario is expected in other cancers displaying aberrant PKCα upregulation. For example, in TNBC, PKCα plays a major role in the self-renewal and tumor-initiating capacities of cancer stem cells, and a significant correlation between PKCα expression and stemness signatures has been observed (138, 139). Notably, PKCα controls the levels and activation of transcription factors/regulators STAT3, FRA1, TAZ, and SMAD3, as well as pluripotent transcription factors Oct4 and Nanog (138, 139, 140, 141), underscoring a potential DAG-regulated control of stemness transcriptional networks *via* this cPKC.

### PKC isozymes as negative regulators of proliferation and tumorigenesis

Besides the described positive links between PKCs and mitogenesis/tumorigenesis, there is ample evidence for inhibitory roles for PKC isozymes in these settings, including various examples causally associated with the control of transcriptional events. One paradigmatic example is represented by the negative regulation of cell cycle by PKCα in intestinal cells. Jennifer Black’s laboratory found that PKCα plays a fundamental role in the maintenance of intestinal homeostasis, being primarily cytosolic (*i.e.*, inactive) in proliferating epithelial cells of intestinal and colonic crypts, and plasma membrane associated (*i.e.*, activated) in growth arrested cells in the midupper crypts. PKCα coordinates signaling pathways leading to cell cycle withdrawal *via* induction of cyclin-dependent kinase inhibitors p21 and p27, as well as cyclin D1 downregulation (142). A key mechanistic step in this cell cycle arrest is the impaired cyclin D1 translation—that is, a posttranscriptional event—by sustained PKCα activation that involves hypophosphorylation/activation of the translational suppressor 4E-binding protein 1, leading to sequestration of cyclin D1 mRNA in 4E-BP1–associated complexes (143, 144). Additionally, a PKCα-driven cascade regulating cyclin D1 gene expression in intestinal cells takes place *via* inhibitor of DNA binding 1 (Id1), a transcriptional regulator that inhibits the transcriptional activity of basic helix-loop-helix transcription factors and whose expression in the intestine is restricted to proliferating crypt cells (145). A comprehensive molecular analysis revealed that PKCα forms a complex with Raf kinases that dissociates upon PKCα activation. The resulting prolonged activation of the ERK pathway confers antiproliferative signaling and involves the cooperation between p21 upregulation (mediated by the Ras-guanine nucleotide exchange factor RasGRP3 and H-Ras) and Id1 downregulation, which results in reduced cyclin D1 levels (146). While the specific phosphorylation events governing Id1 expression *via* the PKCα–ERK pathway are yet to be disentangled, it has been well established that Id transcription factors are heavily regulated by phosphorylation (147), predicting that PKCα repression of Id function/stability is likely dependent on a PKCα-mediated posttranslational modification. Another potential mechanistic link involves c-Myc, a major regulator of Id1 induction that is repressed upon PKCα activation (148, 149, 150).

The growth suppressing role of PKCα has been extended to models of colon cancer. Quite remarkably, Apc^Min/+^ mice develop more aggressive intestinal tumors in a PKCα-deficient background (151). Mechanistic analysis revealed that PKCα suppresses colon cancer cell proliferation by downregulating β-catenin, likely controlling the expression of Tcf/β-catenin–dependent transcription of gene targets that play important roles in cell cycle progression and tumorigenesis, namely cyclin D1, c-myc, and Wnt-specific genes. A model has been postulated in which PKCα mediates N-terminus phosphorylation of β-catenin, which marks it for proteasomal degradation, as well as phosphorylation of the nuclear receptor retinoic acid–related orphan receptor (RORα) that leads to inactivation of β-catenin cotranscriptional activity (152, 153, 154). This paradigm mechanistically explains the tumor suppressor effect of PKCα in colon tumors that display accumulation of β-catenin, and it is also consistent with the fact that in the normal intestinal epithelium, nuclear β-catenin is only detected in the proliferating lower crypts (142). Thus, activated PKCα in the midupper intestinal crypts may also contribute to the repression of β-catenin–mediated transcription.

Another PKC negatively associated with tumor growth is PKCδ. In addition to the PKCδ-mediated apoptotic effect described above, there is unwavering evidence for an antiproliferative action of PKCδ both in normal and transformed cells. We previously reported that phorbol ester-induced G1 arrest in lung adenocarcinoma cells is selectively mediated by PKCδ-dependent induction of p21 without inhibition of cyclin D1 expression, however with a major inhibition of Rb-dependent cyclin A promoter activity (40). Irreversible G2/M cell cycle arrest occurs when the PKC activator is added in S phase, which results in a senescent phenotype, in this case mediated by PKCα induction of p21 (39). While the complexities of PKCδ effector signaling players regulating transcriptional networks are yet poorly understood, studies have linked this nPKC to suppressed proliferation through the action of Kruppel-like factor 4 (KLF4), NF-κB, and β-catenin/TCF transcription factors among others (155, 156, 157, 158, 159). Based on the remarkable effect that PKCδ exerts on the control of transcriptional networks (48), it is predicted that PKCδ has profound effects on the function and/or relocalization of transcription factors.

## PKC-regulated nuclear events and the control of differentiation

The process by which cells mature to acquire specific shapes and functions, including the stepwise differentiation of pluripotent stem cells toward specific cell types and the transition into late differentiated lineages, is tightly dependent on the activation and repression of select transcriptional programs. Genetic, molecular, and pharmacological evidence has shed light into the complex signals impacting the spatiotemporal regulation of transcriptional events governing differentiation, including the identification of ligand/receptors and their signaling effectors triggering the differentiation process. As pleiotropic kinases capable of phosphorylating multitude of intracellular substrates, PKC isozymes play prominent roles in cell differentiation in an assortment of biological systems (Table 1). In fact, PKC modulatory compounds have been early recognized as differentiation agents particularly in epidermal and hematopoietic cells (160, 161, 162). This set the basis for studies on the involvement of PKC isozymes in processes of normal differentiation *via* regulation of transcription factors, as well as illuminated the potential therapeutic targeting of these kinases for diseases such as cancer.

**Table 1:** PKC isozymes in cell lineage differentiation

Lineage	PKC isozyme	Transcription factor
Epidermal	PKCη	c-fos/c-jun (AP-1), C/EBPα, HOXA7
	PKCα	DLX3
	PKCδ	KLF4
Mesenchymal		
Osteogenic	PKCδ	RUNX2
Adipogenic	PKCδ	RUNX2
Myogenic (cardiac)	PKCε	Nkx2.5, GATA-4
Myogenic (skeletal)	PKCε	MyoD TFs, Nrf2
Hematopoietic		
Myeloid (erythrocytic)	PKCα	GATA-1-2
	PKCθ	EKLF
Myeloid (megakaryocytic)	PKCε	GATA-1
Myeloid (dendritic cell)	PKCβ	?
	PKCδ	?
	PKCε	?
Lymphoid (T cell)	PKCθ	AP-1, NF-κB, RORγt, FoxO1, Fox3A
Lymphoid (B cell)	PKCβ	?
	PKCδ	?

Abbreviations: AP-1, activator protein-1;DLX3, distal-less homeobox 3;EKLF, erythroid krüppel–like factor; KLF4, Kruppel-like factor 4; ROR, retinoic acid–related orphan receptor; TF, transcription factor.

Selected members of the PKC family play important modulatory roles in differentiation. The table depicts the most representative examples of PKC isozyme-regulation of transcription factors leading to differentiation of epidermal, mesenchymal, and hematopoietic cell lineages.

### Transcriptional regulation of epidermal differentiation by PKC

Pioneer studies by Yuspa, Blumberg, and others established calcium and DAG as essential signals for epidermal differentiation *via* PKC isozymes (162, 163, 164, 165, 166). PKCs turned out to be important regulators of the coordinated changes in gene expression that occur during keratinocyte differentiation. Notably, phorbol ester treatment of keratinocytes in culture induces the formation of cornified envelopes, a highly insoluble structure formed beneath the plasma membrane that is composed of specific precursor proteins cross-linked by the action of transglutaminases during keratinocyte terminal differentiation. PMA is a prominent inducer of *TGM1*, the transglutaminase 1 gene, in primary mouse epidermal cells (162, 167, 168). Interestingly, calcium-induced keratinocyte differentiation is associated with changes in the expression of selected PKC isozymes, specifically a reduction in PKCα and a concomitant elevation in PKCε and PKCη, suggesting PKC isozyme selective roles at different stages of epidermal differentiation. A redistribution of specific PKCs to the membrane during keratinocyte differentiation likely reflects their activation status (165). PKCη, a PKC highly expressed in skin, was identified as a crucial mediator for the transcriptional activation of *TGM1*, as well as for other genes coding cornified envelope structural proteins such as involucrin and loricrin (169, 170, 171).

While the molecular details underlying PKC-driven transcriptional changes are only partially understood, further studies revealed the engagement of distinctive signaling effectors for different differentiation marker genes. For example, PKCη-mediated transcriptional control of *TGM1* involves Sp1 transcription factors acting in a proximal (−95 to −67) element in the transglutaminase 1 gene promoter (172). Additional roles for c-fos/c-jun and the transcription factor HOXA7 have been identified in the control of *TGM1* expression in keratinocytes (173, 174), although the identity of the PKC isoform involved in this case remains to be defined. Activation of PKCη induces the expression of the differentiation transcription factor C/EBPα and its binding to the involucrin gene promoter through a cascade that involves the Ras/p38 MAPK pathway (175). An extensive analysis of the gene coding for cornifin-A (*SPRR1A*), a cross-linked envelope protein of keratinocytes, identified a ∼170 base pair region in the proximal promoter region with a single AP-1 binding site and two Ets binding sites that were necessary for the regulated gene expression by calcium or PMA (176). In addition to the established relevance of AP-1 in keratinocyte differentiation transcriptional programs, most recent studies identified molecular regulatory events driven by KLF4, distal-less homeobox 3 (DLX3), and E2F1, with selective dependence on PKC isozyme specificity and differentiation stimulus (155, 156, 177, 178). An interesting case reflecting the intricate PKC signaling network is the fibroblast growth factor receptor 2–induced keratinocyte differentiation, wherein PKCδ controls early differentiation steps and PKCα regulates terminal stages, acting respectively through the induction of KLF4 and DLX3 transcription factors (156). Still, we are far from attaining a comprehensive picture of the PKC-regulated transcriptome associated with epidermal differentiation.

### PKC and mesenchymal stem cell differentiation

The differentiation of mesenchymal stem cells (MSCs) into specific lineages—osteogenic, chondrogenic, myogenic, adipogenic—is largely influenced by phosphorylation mechanisms, both in positive and negative manners (179). The RUNX family of transcription factors (RUNX1, RUNX2, and RUNX3) plays essential roles in normal development by controlling differentiation and cell lineage specification. They are part of heterodimeric complexes between the main partner (RUNX) and the core binding factor subunit β. RUNX transcription factors could act both as transcriptional activators or repressors, and they can be profoundly regulated by posttranslational modifications, including phosphorylation (180, 181). In this context, the contribution of a PKC–RUNX2 pathway to skeletal development represents a well-characterized mechanism. RUNX2 is a master regulator for the *de novo* bone formation, a process involving MSC differentiation both into osteoblasts (which mature into osteocytes to form bone, that is, intramembranous ossification) and into chondrocytes (which form permanent cartilages or alternatively cartilages that mature to bone, that is, endochondral ossification) (182). RUNX2 is a downstream target of numerous osteogenic factors, including fibroblast growth factor 2 (FGF2), the Notch ligand Jagged-1 (Jag1), Wnt ligands (*e.g.*, Wnt3a) and glucagon-like peptide-1 (180, 181, 182, 183, 184, 185). Notably, pharmacological inhibition of PKC in a preosteoblastic cell line not only prevents RUNX2 upregulation induced by FGF2 but also impairs RUNX2 binding activity and transactivation function (186). Pathway analysis revealed the involvement of PLCγ1 and PKCδ as crucial mediators of the FGF2 signaling response. Interestingly, PKCδ physically associates with RUNX2 after FGF2 stimulation, an interaction that requires the translocation of PKCδ to the nucleus. PKCδ nuclear relocalization and its regulatory action on RUNX2 is greatly enhanced by connexin-43, a gap junction protein that is abundantly expressed in osteoblasts and required for osteogenic activity in response to extracellular cues (187, 188, 189).

PKCδ is also essential for osteoblast differentiation by the Notch ligand Jag1. Jag1 triggers a rapid translocation of PKCδ to the nucleus and its activation in osteoblast precursors. In this signaling context, PKCδ activation is mediated by the Notch intracellular domain (NICD) that results from receptor cleavage. Notably, NICD associates with PKCδ in response to Jag1 stimulation. It has been hypothesized that PKCδ-mediated phosphorylation of NICD prevents the proteasomal degradation of this receptor fragment, ultimately favoring NICD nuclear relocalization and the subsequent activation of Notch transcriptional targets (190). Thus, PKCδ acts both in the nucleus by promoting RUNX2 activation and in the cytosol by upholding NICD stabilization (*i.e.*, stimulation of canonical Notch transcriptional activation). This dual mechanism ultimately provides a strong signaling input for Notch receptor–mediated transcriptional activation of genes associated with osteoblastogenesis. Consistent with this model, osteogenic lineage commitment of human bone marrow–derived MSCs is reduced upon pharmacological inhibition or genetic ablation of PKCδ. This pathway has also been recognized as important in adipogenic differentiation (191).

PKCε has been implicated in skeletal and cardiac muscle differentiation. PKCε levels along the process of bone marrow mesenchymal cell differentiation follow an opposite pattern than the expression of nkx2.5 and GATA-4, key cardiac differentiation transcription factors. There is a causal relationship between these two events since PKCε negatively regulates the expression of these transcription factors *via* the MEK/ERK pathway (192). Conversely, studies using muscle stem/progenitor cells, myosatellite or satellite cells, implied PKCε as a positive modulator of skeletal muscle differentiation. Most notably, it has been shown that the PKCε inhibitor εV1-2 markedly reduces skeletal muscle cell differentiation. PKCε expression and its nuclear localization are augmented during this differentiation process, leading to the regulation of specific basic helix-loop-helix MyoD myogenic transcription factors. Indeed, PKCε upregulation correlates with changes in the expression of *Mrf4* and *myogenin*, myogenic genes implicated in intermediate/late phases of skeletal muscle differentiation, without affecting the expression of early differentiation genes *MyoD* and *Myf5*. High mobility group A1, a non-histone chromatin associated protein, is downregulated by PKCε, an effect that facilitates *Mrf4* and *myogenin* expression required for activation of the skeletal muscle differentiation program (193). Additionally, PKCε forms a complex with the transcription factor Nrf2, resulting in a sustained expression of the antioxidant enzyme SOD2. This last mechanism leads to a reduction in reactive oxygen species that is required for late stages of differentiation into myotubes (194).

### PKC regulation of hematopoietic cell differentiation

PKC has been widely studied as a signaling player in physiological hematopoietic lineage differentiation. As predicted from the distinctive expression of PKC isozymes and their signaling targets in discrete hematopoietic lineages, unique mechanistic features are involved in PKC-regulated erythroid and myeloid normal differentiation. In addition, PKC signaling has been largely implicated in the aberrant differentiation of hematopoietic cancer cells. Most notably, PKC activators such as phorbol esters, bryostatins, or prostratin have been thoroughly studied as differentiation agents particularly in leukemic cells (160, 161, 195, 196, 197, 198).

Striking variations in the patterns of PKC isozyme expression occur along the differentiation of progenitor pluripotent hematopoietic stem cells to different subsets of lineages. An early study using Q-PCR revealed that in comparison to bone marrow CD34+ lymphohematopoietic stem cells, there is significant up-regulation of PKCα, β, δ, θ, and ι in megakaryocytes and platelets; upregulation of PKCα, β, δ, and ζ in granulocytes and monocytes; as well as upregulation of PKCθ and ι in erythroid cells (199). *In situ* immunofluorescence analysis comparing human progenitor cells with erythroid and megakaryocytic cells, obtained by treatment with erythropoietin (EPO) and thrombopoietin, respectively, confirmed the elevation of cPKCs in megakaryocytes, although a weaker expression cPKC isozymes was found in erythroid cells. Notably, increased nuclear levels of PKCδ and PKCζ were detected along the differentiation to erythroid and megakaryocytic cells (200). In several cases, the reported changes in PKC isozyme expression/localization have been causally associated with specific lineage commitments. For example, commitment to the macrophage lineage is associated with the translocation of PKCα to the nucleus. Indeed, a catalytically active PKCα mutant, which localizes primarily to the nucleus upon expression in hematopoietic progenitors, mimics the differentiation signal of macrophage colony–stimulating factor (201). PKCα is also required for EPO-induced erythroid differentiation of CD34+ progenitor cells, and it becomes upregulated in response to an erythroid differentiating agent. Notably, specific downregulation of PKCα (but not PKCβII) using a specific ribozyme confers resistance to EPO-induced erythroid differentiation of CD34+ cells, thus establishing a stringent requirement for this kinase (202). Successful megakaryocytopoiesis requires a tight timely control of PKCε expression, together with persistently high levels of PKCδ as a requisite for megakaryocyte maturation and platelet production (203, 204). Notably, PKCδ expression was found to be elevated during megakaryocyte differentiation, and PKCδ−/− mice have reduced circulating platelet count (205). PKCθ is crucial for the positive selection of thymocytes, the maturation of naïve T cells to mature T lymphocytes, and the commitment to T helper (Th) 17 cells, a function shared with PKCε (206, 207, 208, 209, 210, 211), whereas PKCβ, PKCδ, and PKCε have been causally linked to progenitor cell differentiation to dendritic cells (212, 213, 214). Evidence has been also provided for the involvement of PKCβ and PKCδ in B lymphocyte differentiation and cell fate (215, 216).

Considering the complexities of the transcriptional networks controlling hematopoietic cell differentiation, it is not surprising that individual PKCs modulate specific nuclear events depending on lineages and stages of differentiation, most of which remains poorly comprehended. For the EPO-induced erythroid lineage differentiation, PKC-dependent increase in GATA-2, a transcription factor critical for the maintenance of immature hematopoietic progenitors, has been observed (217). Additionally, PKCα controls the expression of GATA-1, a crucial transcription factor that activates genes driving erythroblast maturation to erythrocytes. A study in zebrafish revealed that PKCα interacts with and phosphorylates the mRNA stabilizing protein ELAV1/HuR in Ser219 and Ser 316, leading to ELAV1/HuR nucleocytoplasmic shuttling and stabilization of GATA-1 mRNA *via* binding to AU-enrichment elements in the 3′-UTR. This ultimately leads to increased GATA-1 translation in polysomes that bolsters erythropoiesis (218). A second well-characterized mechanism in erythroid lineage differentiation relates to the tight regulation of erythroid krüppel–like factor (EKLF) nuclear import by PKCθ, particularly during the transition from proerythroblast to basophilic erythroblast. The transcription factor EKLF redistributes to the nucleus in a PKCθ-dependent manner, a step that depends on PKCθ-mediated phosphorylation at Ser68 in EKLF that leads to its dissociation from its cytoplasmic partner “Foe of EKLF.” EKLF phosphorylation by PKCθ also enhances its sumoylation, contributing to the interaction of the transcription factor with importin β1 and thus enabling its nuclear import. This combined mechanism is critical for the committed erythroid progenitors to enter an irreversible terminal differentiation process (219).

PKC-mediated regulation of the GATA-1 transcription factor also plays a role in megakaryopoiesis, although unlike PKCα-driven erythroid differentiation, here the PKCε isoform turned out to be the major player. It has been hypothesized that PKCε targets component(s) of the transcriptional GATA-1 machinery related to megakaryopoiesis. For example, constitutively active PKCε mutants, but not mutants for other PKCs, cooperate with GATA-1 in the activation of the megakaryocytic-specific αIIb integrin promoter. Consistent with this finding, megakaryocytic differentiation is impaired by the pan-PKC inhibitor GF109203X but not the cPKC inhibitor Gö6976, further supporting the involvement of this nPKC in this process (220). PKCε has been found to localize to the nucleus in a myelogenous leukemia cell line, suggesting potential phosphorylation of transcription factors or transcriptional modulators by this kinase (220). Nonetheless, there is an overall limited mechanistic understanding of the nuclear mechanisms regulated by PKC isozymes along the commitment to megakaryocytes/platelets or other myeloid progenitor–derived cell populations, that is, monocytes and monocyte-derived cells (macrophages, dendritic cells), and granulocytes. Specific links between individual PKCs and transcription factors such as AP-1, NF-κB for these myeloid-derived lineages have been reported (221, 222, 223, 224), but the mechanistic insights and functional implications are yet to be unraveled.

As described above, PKCθ has been established as the main PKC controlling T lymphocyte function and as a key downstream TCR effector. PKCθ acts as an essential mediator of TCR-driven T-cell activation and proliferation, mechanisms that involve the transcriptional activation of genes encoding for IL-2 and other mediators and that depend primarily on AP-1 and NF-κB transcription factors (206, 225). Beyond these crucial roles, PKCθ also exerts an important modulatory control on T lymphoid lineage differentiation. Using PKCθ-deficient mice, the Altman laboratory revealed that this kinase is required for T cell survival and the differentiation into fully competent cytokine-producing cytotoxic T lymphocytes (226). PKCθ also turned out to be critical for the development of Th2 cells and the expression control of GATA-3, a master Th2 transcription factor, thereby regulating the production of key cytokines in this lineage (227, 228, 229). Notably, overexpression of GATA-3 in PKCθ-deficient Th2 cells increases the production of Th2 cytokines, including IL-4, IL-5, IL-10, IL-13, and IL-24 (227). A PKCθ/NF-κB pathway has been also established as an important step for the polarization of naïve CD4+ cells into Th2 cells and Th2 cytokine production (208, 230). Additionally, PKCθ plays a key role in regulating differentiation into proinflammatory Th17 cells. This involves the integration of multiple transcriptional events, namely the dependence on cyclic AMP response element binding protein, AP-1, and NF-κB transcription factors (208, 231). In the context of Th17 differentiation, the steroid receptor coactivator SRC1 has been identified as a PKCθ substrate. Phosphorylation on Ser 1271/1272 modulates SRC1 ability to bind and activate RORγt (shorter isoform), a transcription factor required for Th17 differentiation. Additionally, phosphorylated SRC1 overcomes Foxp3-mediated inhibition of RORγt by SRC1 and controls epigenetic events that open the *IL17* locus for optimal transcription (210). In Tregs, cells that control immune responses and maintain self-tolerance, PKCθ is sequestered away from the IS. Unlike in effector T cells, PKCθ in Regulatory T cells inhibits Treg-mediated suppressive function (232). Interestingly, PKCθ-mediated signaling inhibits the differentiation of naïve T cells into inducible Tregs. PKCθ−/− T cells have increased Treg differentiation capacity *in vitro*, an effect that is mediated by Akt and the FoxO1/FoxO3A transcription factors (233). Considering the recently identified PKCθ-mediated phosphorylation events that control alternative splicing mechanisms in Tregs (234, 235), it is evident that this kinase orchestrates several epigenetic and transcriptional responses that establish phenotypic dominance of Th17 over the Treg pathway.

## PKC regulation of EMT transcription factors

Epithelial-to-mesenchymal transition (EMT) is a process by which epithelial cells undergo biochemical and phenotypical changes to attain a mesenchymal phenotype, including enhanced migratory and invasive capacities. This phenotypic plasticity that takes place both during normal physiological events (*e.g.*, organ development), wound healing, tissue regeneration, and pathological processes (*e.g.*, cancer progression and metastasis) is triggered by extracellular signals such as transforming growth factor-β, growth factors, and cytokines among others. Key to EMT is the activation of specific transcription factors, production of extracellular matrix-degrading proteins, and reorganization of the cell cytoskeleton, events that are all heavily regulated by PKCs (22, 49, 236, 237, 238, 239, 240, 241).

While at the present time there is no unanimous consensus for a functional association between PKC and EMT, there has been speculation that discrete PKCs may play important roles in initiating EMT and/or maintaining the mesenchymal phenotype (22, 121, 139, 241, 242, 243, 244, 245). Most importantly, the current evidence supports the involvement of PKC isozymes both in the control of EMT transcription factor expression/activity as well as in the regulation of EMT transcriptional networks, particularly in cancer (22, 71, 135, 139, 246). A salient paradigm is the prominent control of EMT transcription factors exerted by PKCα, as exemplified for Zeb1 in TNBC (247, 248) and hepatocellular cancer (249). PKCα also phosphorylates Twist1 to prevent the degradation of this EMT transcription factor *via* ubiquitination (250). Additionally, PKCα mediates Snail1 up-regulation induced by transforming growth factor-β through a pathway that involves ERK (251).

**Figure 4:**
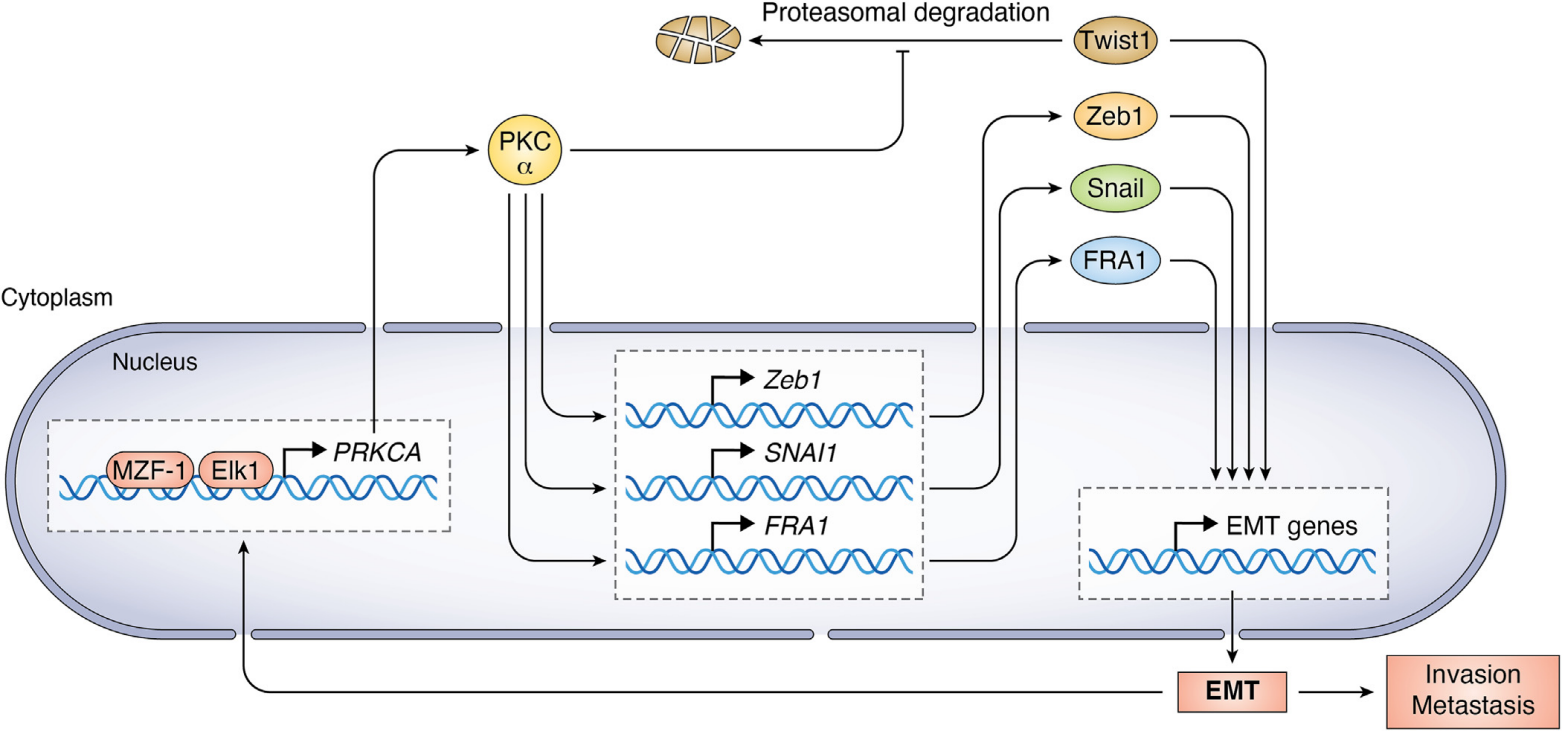
**PKCα and the control of EMT.** PKCα is up regulated in mesenchymally transformed cells in TNBC and likely in other cancer types. Transcriptional deregulation of the PKCα gene (*PRKCA*) in EMT is mediated by MZF-1 and Elk-1 transcription factor complexes. PKCα controls the expression of selected EMT transcription factors by promoting transcriptional activation of their corresponding genes or by enhancing their stability, thus leading to a vicious cycle for cancer cell invasiveness. Elk-1, Ets-like protein-1; EMT, epithelial-to-mesenchymal transition; triple-negative breast cancer; MZF-1, myeloid zinc finger-1.

A pivotal study by the Weinberg laboratory reported that inhibition of PKCα specifically targets breast cancer cells that have undergone EMT and are enriched for stem cell properties. There is a causal association between high levels of PKCα and the expression of the transcription factor FRA1 in TNBC. Remarkably, inhibition of the PKCα-FRA1 link leads to reversion of the mesenchymal phenotype (*i.e.*, mesenchymal-to-epithelial transition) (139). The elevated expression of PKCα observed in mesenchymally transformed TNBC cells is driven by transcriptional deregulation of the PKCα gene (*PRKCA*) by a myeloid zinc finger-1 and Ets-like protein-1 transcription factor complex (252). In this manner, a convoluted network of transcriptional events perpetuates the mesenchymal phenotype driven by PKCα (Fig. 4). Together with the reported nontranscriptional EMT effects mediated by this PKC, such as stabilization of E-cadherin at adherent junctions (253), these findings have significant therapeutic implications, since inhibition of PKCα-mediated signaling may reverse the mesenchymal phenotype and ultimately sensitize cancer cells to therapy (139, 246, 252, 254, 255). It is expected that these multifaceted mechanisms would be further elucidated in the years to come, which would rationalize the potential use of PKC isozymes and their effectors as biomarkers and targets for metastatic disease.

## A case for the atypical PKCs in the control of nuclear function

aPKCs ζ and ι, the DAG/calcium unresponsive PKC isozymes, are regulated through a complex set of interactions with proteins and lipids. Their distinctive structural features that include a single DAG/phorbol ester C1 domain, the absence of a C2 domain, and unique protein–protein interaction motifs, confer characteristic regulatory modes that do not involve DAG and calcium as enzyme activators (256, 257). The pleiotropic cellular roles of aPKCs in cell biology include major regulatory effects on cell polarity, proliferation, survival, and stemness among others. Not surprisingly, exquisite roles in tumorigenesis have been attributed to aPKCs in different contexts, most generally as a tumor promoter for PKCι and as a tumor suppressor for PKCζ, as detailed in excellent reviews by the Moscat/Diaz-Meco group (256, 258, 259, 260).

The involvement of aPKCs in cell signaling uncovered a few effector pathways impacting nuclear regulatory function. The best studied example is the PKCζ-NF-κB link that has been associated with a range of effects, including the modulation of cytokine production, cell proliferation and survival, cancer cell invasiveness, tumor growth, and inflammatory responses, to name a few (258). Mechanistically, the postulated model is that PKCζ phosphorylates Ser311 in RelA (p65) after the release from its complex with the IκB inhibitory protein, a modification that is required for full NF-κB transcriptional activation. Phosphorylation of Ser311 facilitates the recruitment of the CBP/p300 coactivator complex that allows Lys310 acetylation, thereby promoting the opening of chromatin and transcriptional activation of NF-κB target genes. Key activating phosphorylating events by PKCζ on IκB kinase also suggest its contribution to NF-κB upstream events (258, 261). Several aPKC-mediated transcriptional responses, including those related to cell proliferation, have been also linked to modulation of the ERK, β-catenin, and STAT pathways (262, 263, 264, 265, 266, 267). PKCζ has been also shown to associate with the nuclear factor of activated T cells transcription factor in T cells, leading to its phosphorylation and enhanced transcriptional activation (268).

A transcriptomic profiling of PKCι-depleted prostate epithelial cells revealed prominent cell cycle–related gene profiles, as well as E2F, Myc, and Mammalian target of rapamycin complex 1 signatures. In addition, important changes in genetic signatures associated with the unfolded protein response and Ser/Gly biosynthesis have been identified in neuroendocrine prostate cancer models (269). PKCι also phosphorylates the FoxO1 transcription factor on Ser218, limiting its DNA binding ability and affecting the expression of target genes such as c-Myc (270). A complex picture has also been established in aPKC control of differentiation programs. Interestingly, despite the reported expression/localization changes of PKCζ along hematopoietic differentiation (see above), mice deficient of PKCζ and PKCι have normal hematopoiesis, including normal hematopoietic stem cell renewal and differentiation into myeloid and lymphoid lineages (271). While this may be related to lack of involvement of aPKCs in polarity signaling during stem cell fate determination, it also denotes the absence of relevant links with hematopoietic differentiation transcriptional events. Still, crucial roles for aPKCs in cell fate decisions have been reported, such as the PKCζ-mediated GATA3-dependent mitotic spindle orientation required for lineage specification from prostate progenitor cells (272) and the PKCι-dependent differentiation of trophoblast progenitors into multinucleated syncytiotrophoblast cells during placentation *via* Glial cells missing transcription factor 1, GATA binding protein 2, and Peroxysome proliferator-activating receptor transcription factors (273). PKCι was found to be required for Paneth cell differentiation in the intestine. In this case, loss of PKCι increases the expression of EZH2, a component of the transcriptional repressor complex polycomb repressor complex 2, which results in repression of atonal homolog 1 and growth factor independent 1, critical transcription factors for the differentiation of Paneth cells (274). This heterogeneity highlights the potential context-dependent roles for aPKCs in differentiation processes.

Work from Alan Fields and co-workers postulated PKCι as a tumor promoting kinase acting through a unique mechanism that depends on rRNA synthesis in the nucleolus (275). Key to its tumorigenic activity is the aberrant amplification of the *PRKCI* gene, a hallmark of ovarian and lung cancer (257, 265, 276, 277, 278), resulting in deregulated activation of the guanine nucleotide exchange factor Ect2 and activation of the small GTPase Rac1 in the nucleus. Nuclear phosphorylation of Ect2 by PKCι on Thr328 stimulates the synthesis of rRNA, the major component of ribosomes, in lung adenocarcinoma cells. This phosphorylation event promotes the formation of an Ect2 complex with upstream binding factor-1 (the major rDNA transcription factor) in the nucleolus and enables the recruitment of Rac1 and nucleophosmin (a nucleolar protein that binds rDNA promoters and remodels ribosomal chromatin) to activate rDNA transcription. The resulting activation of nucleolar rRNA synthesis *via* this PKCι-driven mechanism is critical to fuel protein synthesis required to sustain the excessive proliferative capacity of transformed cells (275, 279, 280). Targeting this nuclear pathway could indeed represent an attractive target for therapeutic intervention in cancer.

## Concluding remarks

PKC isozymes function as pleiotropic kinases that oversee multitude of signaling pathways, many of them impacting on nuclear function. As fundamental signaling nodes, PKCs control transcriptional networks relevant for normal cellular homoeostasis, including cell proliferation, motility, polarity, and differentiation. Individual PKC isozymes distinctively regulate the activity of signaling pathways leading to the transcriptional activation or repression of genes either by direct phosphorylation of transcriptional complex components or *via* modulation of downstream cascades that impinge on gene expression control. The control of cell cycle and normal lineage differentiation represent some of the best examples in which PKC isozymes, in a strictly cell type–dependent context, regulate essential cellular functions *via* nuclear pathways. Aside from the physiological roles of PKC isozymes in normal cells, aberrant PKC signaling in pathological stages, either by deregulated expression or activation, promotes anomalous phosphorylation of downstream effectors, leading to profound alterations in gene expression and the potential rewiring of transcriptional networks. These alterations are very common in cancer, where aberrant activation of discrete PKCs largely impacts transcriptional profiles associated with mitogenesis, invasiveness, and the tumor microenvironment. Dissecting the complexities of PKC isozyme-specific nuclear regulation would allow a better comprehension of their gene effectors in pathophysiology and pave the way for the identification of molecular targets for a wide spectrum of diseases.

## Conflict of interest

The authors declare that they have no conflicts of interest with the contents of this article.
